# Single-cell and microarray chip analysis revealed the underlying pathogenesis of ulcerative colitis and validated model genes in diagnosis and drug response

**DOI:** 10.1007/s13577-022-00801-6

**Published:** 2022-11-29

**Authors:** Liqing Yang, Haiying Chen, Yunong Yang, Yeling Deng, Qiumin Chen, Baiwei Luo, Keren Chen

**Affiliations:** 1 Central People’s Hospital of Zhanjiang, Zhanjiang City, Guangdong Province China; 2 The First Clinical Medical College, Guangdong Medical University, Zhanjiang City, 524000 China

**Keywords:** scRNA-seq, Ulcerative colitis, Diagnosis model, Anti-TNFα therapy, Inflammatory bowel disease

## Abstract

The morbidity rate of ulcerative colitis (UC) in the world is increasing year by year, recurrent episodes of diarrhea, mucopurulent and bloody stools, and abdominal pain are the main symptoms, reducing the quality of life of the patient and affecting the productivity of the society. In this study, we sought to develop robust diagnostic biomarkers for UC, to uncover potential targets for anti-TNF-ɑ drugs, and to investigate their associated pathway mechanisms. We collected single-cell expression profile data from 9 UC or healthy samples and performed cell annotation and cell communication analysis. Revealing the possible pathogenesis of ulcerative colitis by Gene Ontology (GO), Kyoto Encyclopedia of Genes and Genomes (KEGG), and Gene Set Enrichment Analysis (GSEA) analysis. Based on the disease-related modules obtained from weighted correlation network analysis (WGCNA) analysis, we used Lasso regression analysis and random forest algorithm to identify the genes with the greatest impact on disease (EPB41L3, HSD17B3, NDRG1, PDIA5, TRPV3) and further validated the diagnostic value of the model genes by various means. To further explore the relationship and mechanism between model genes and drug sensitivity, we collected gene expression profiles of 185 UC patients before receiving anti-tumor necrosis factor drugs, and we performed functional analysis based on the results of differential analysis between NR tissues and R tissues, and used single-sample GSEA (ssGSEA) and CIBERSORT algorithms to explore the important role of immune microenvironment on drug sensitivity. The results suggest that our model is not only helpful in aiding diagnosis, but also has implications for predicting drug efficacy; in addition, model genes may influence drug sensitivity by affecting immune cells. We suggest that this study has developed a diagnostic model with higher specificity and sensitivity, and also provides suggestions for clinical administration and drug efficacy prediction.

**Supplementary Information:**

The online version contains supplementary material available at 10.1007/s13577-022-00801-6.

## Introduction

Ulcerative colitis, a chronic inflammatory disease affecting the colon, is frequently recurrent. The incidence of ulcerative colitis is on the rise globally, with an incidence of 2.2–14.3 cases per 100,000 people per year and a prevalence of 37–246 cases per 100,000 people per year in North America, and an increasing incidence in developing countries [1, 2]. Recurrent episodes of diarrhea, mucopurulent blood in the stool, and abdominal pain are the main symptoms, impacting the quality of life of patients and the productivity of society.

Endoscopy is still the main diagnostic method for UC, but imaging examinations cannot detect early lesion that make it difficult to diagnose in early stage. In addition, although some laboratories have identified some biomarkers for monitoring the inflammatory process, they are not suitable for establishing new diagnoses. Therefore, its an urgent for us to find biomarkers for diagnosis of UC to diagnose UC as soon as possible. At present, treatment of mild to moderate ulcerative colitis involves, primarily, 5-aminosalicylates, while thiopurines or biological agents (anti-tumor necrosis factor alpha or anti-integrin therapy) are used for moderate to severe disease. However, even with medical therapy, 15% of patients require will surgery to treat UC or disease-associated complications of dysplasia [3, 4]. Here raises the question of differential response to anti-TNF-α treatment. However, due to imprecision in predicting the response to anti-tumor necrosis factor alpha agents, patients must often receive long-term therapy to determine if the drug of choice is effective, which often delays the diagnosis and initiation of appropriate treatment. Therefore, it is important to effectively predict the responsiveness of UC patients to anti-TNF-α.

In this study, we focused on finding diagnostic biomarkers of UC, revealing its pathogenesis, and exploring potential mechanisms of anti-TNF-ɑ drug sensitivity in UC patients. With the rapid development of high-throughput technology and single-cell technology [5], more technologies have been developed for research. The application of single-cell technology in UC has also been reported [6] and recently, an increasing number of researchers have used the developing high-throughput technology to diagnose diseases or predict drug response, thereby, providing opportunities for treatment. Therefore, a comprehensive comparison of genetics, gene expression data, and microorganisms provides an excellent opportunity to study the molecular mechanisms of resistance to anti-tumor necrosis factor alpha drugs and predictive biomarkers. In this study, we sought to use these techniques to develop a robust diagnostic profile of UC and reveal relevant regulatory mechanisms, which may help to elucidate its pathogenic mechanisms, identify valuable diagnostic biomarkers for UC, and contribute to the search for biomarkers and potential targets for early diagnosis, prevention, and treatment of related diseases.

## Materials and methods

### Data collection and pre-processing

We obtained raw data for transcriptome analysis of 9 single-cell samples from GSE116222 through the Gene Expression Omnibus (GEO) database (https://www.ncbi.nlm.nih.gov/geo/). Colon biopsies were collected from inflammatory areas of the colon and adjacent non-inflammatory areas in healthy patients (healthy) and patients with UC inflammation. Using the Seurat package (Version 4.0.0), Seurat manipulation objects were created for single-cell data from nine samples, and a total of 11,175 cells were obtained. To reduce the effect of “empty droplets” (i.e., a cell gel containing no cells or cell debris) or “overloaded droplets” (i.e., a cell gel bead containing more than 1 cell). Cells were defined as “eligible cells” if they met the following conditions: (1) the number of genes in the cell was greater than 500 and less than 5000; (2) the percentage of mitochondrial gene expression was less than 5%. After screening, 11,005 eligible cells remained. The single-cell data were normalized using the default method of the NormalizeData function of the Seurat package. scaleData was used to reduce the sources of meaningless variation. The harmony package (Version 1.0) was used to remove potential batch effects (1). In addition, we downloaded the mucosal gene expression profiles GSE75214 and GSE87473 from patients with ulcerative colitis for the identification of uc-related genes.

To explore potential targets of UC patients sensitive to anti-TNF-drugs, we obtained two gene expression profiles from GEO for the UC patient cohort, GSE92415 and GSE12251. 21 of 183 GSE92415 samples had no response information to anti-tumor necrosis factor α drugs and were excluded. The remaining 162 UC samples were used as the training set. An additional cohort GSE12251 was added as the validation set (*n* = 23). The raw Affymetrix data (GSE87473, GSE75214, GSE12251, GSE92415) in CEL format were read using Affy [7] and normalized using the robust microarray analysis (RMA) method [8].

### Cell annotation and cell communication analysis

Cells were annotated according to the CellMarker database of marker genes (2). Cell annotation was performed using the SingleR package (Version 1.4.1) to assist in validating the annotation results. singleR is based on high-purity transcriptome data of different cell types, and single-cell data were annotated to the corresponding cell types by correlation analysis. Cell communication analysis is performed using the CellChat package (Version 1.0.0).

### Analysis of variance

For single-cell data, based on unsupervised clustering, differential analysis of ulcerative colitis tissue versus normal tissue was performed by the DEsingle package (Version 1.10.0). DEsingle uses a zero-inflated negative binomial model to assess the defined differential genes.

### Identification of ulcerative colitis-associated hub genes using WGCNA

WGCNA is a systematic biological method for constructing scale-free networks using gene expression data. Using WGCNA, we were able to cluster genes that show similar expression patterns and identify the modules that are the most relevant for the onset of UC.

We used the WGCNA package to cluster the samples, set the cut tree height to 90 to identify and eliminate outlier samples, selected a soft threshold of 16 to construct a scale-free network, and set the minimum number of genes per module to 30, used the dynamic cut method to cut the tree into different modules, calculated the feature vector of each module in turn, and then clustered the modules, and the modules with similarity over 0.75 on the clustering tree would be merged. After further calculating the correlation between each gene and the module, we selected the gene of the module most relevant to the disease as the hub gene.

### Identifying diagnostic genes and validating their validity

The hub gene was included in the Lasso regression model analysis, which was implemented in the training cohort using the “glmnet” package. The best penalty lambda parameter would be chosen through a tenfold cross-validation [9]. Based on the best detected lambda, we can obtain a list of diagnostically relevant genes with correlation coefficients from gene expression and patient clinical data. The random forest combines several weak classifiers to make the model more accurate. To further identify the genes with the greatest impact on the disease, we used the random forest algorithm, set the number of decision trees to 500, ranked the diagnosis-related genes according to their contribution to the model, and used the top 5 genes as model genes. (EPB41L3, HSD17B3, NDRG1, PDIA5, TRPV3). By analyzing receiver-operating characteristic (ROC) curves, gene sensitivity and specificity were determined. Based on the expression level of each gene weighted by its multivariate LASSO regression coefficient, we calculated the risk score for each patient [RiskScore = (−1.773 * expression level of EPB41L3) + (−1.905 * expression level of HSD17B3) + (−1.818 * expression level of NDRG1) + (3.443 * expression level of PDIA5) + (−0.262 * expression level of TRPV3) + 22.68]. ROC analysis identified a cutoff value of 2.062 for the risk score, as well as a classification of the training cohort by high or low risk. The riskscore is plotted and the heat map is drawn according to the risk score ranking. To assess whether the diagnostic model was more accurate for disease diagnosis than previous studies, we quantified the net reclassification improvement (NRI) [https://doi.org/10.1002/sim.4085] and integrated discrimination improvement (IDI) [https://doi.org/10.1002/sim.5647], and further performed decision curve analysis (DCA) [10] as well as comparing the C-index of the old and new models. NRI and IDI are two quantitative measures of whether a diagnostic model can be improved. It can be defined as the difference in event and non-event prediction probabilities between the old and new diagnostic models. an NRI/IDI > 0 indicates a positive improvement in disease diagnosis by the diagnostic model; an NRI/IDI < 0 indicates a negative improvement, or no improvement when NRI/IDI = 0. DCA shows the net benefit of managing according to the classification system at different adverse event probability thresholds. The larger the area below the decision curve, the more beneficial the classification system is for managing patients who follow it. C-Index has a similar meaning to ROC and ranges from 0 to 1. The closer it is to 1, the greater the predictive value of the model.

### Exploration of single-sample immune cell characteristics

We performed CIBERSORT [11] and ssGSEA [12] analysis of gene expression profiles (GEPs) to compare immune cell characteristics of Non-response (NR) tissues with Response (R)) tissues. ssGSEA allows the application of immune cell population expression characteristics to individual samples and based on the expression profile The rank value of each gene was calculated. The normalized UC GEPs data are compared to the “GSVA” (R package) genome to quantify the relative immune cell infiltration levels of individual samples. To quantify the proportion of immune cells in UC samples, we used the CIBERSORT algorithm, a deconvolution algorithm based on RNA-seq data, to analyze the relative expression of 547 genes in individual tissue samples based on their GEPs and to predict the proportion of 22 immune cells in each tissue.

### Functional analysis

We performed Gene Ontology (GO) analysis, Kyoto Encyclopedia of Genes and Genomes (KEGG) analysis and Gene Set Enrichment Analysis (GSEA) analysis of differential genes using the clusterProfiler package. We considered pathways with *p* values less than 0.05 as statistically significant and included them in the study. The h.all.v7.5.1. symbols were used as the characteristic gene sets for GSEA analysis.

## Results

### Cellular atlas of ulcerative colitis and cellular pathway network

**Fig. 1:**
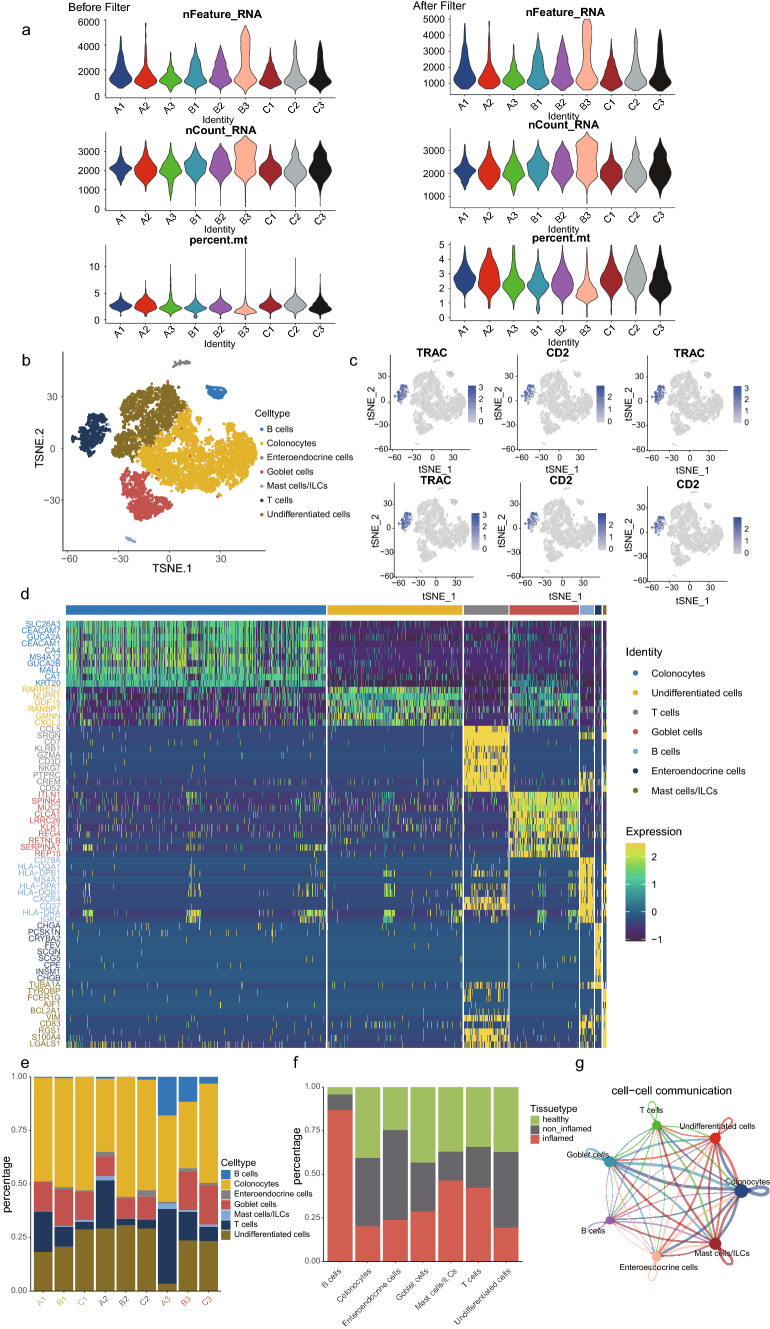
**a** Quality control results for single-cell data. The number of genes, the number of gene reads and the proportion of mitochondrial genes were represented in order from top to bottom. The left panel was pre-processing and the right panel was post-processing. **b** Tsne diagram of cell annotation results based on marker genes and SingleR results. **c** T cell marker genes expression. **d** Heatmap of the top 10 signature genes for different cell types. **e** Expression abundance of different cell types in different samples. **f** Proportions of different cell types in healthy, non-inflamed and inflamed tissues. **g** Interaction network of different cell types, with line thickness indicating the intensity of action and dot size indicating the degree of interaction

After data quality control, low quality cells were filtered out and the number of cells was reduced from 11,175 to 11,005 (Fig. 1a). We used the Seurat package to perform descending and unsupervised clustering analysis of cells, and finally identified 16 cell clusters. The cell populations were annotated according to known marker genes as well as the SingleR package [13], and a total of seven cell types were identified, including B cells, T cells, colonocytes, enteroendocrine cells, goblet cells, mast cells/innate lymphoid cells (ILCs) and undifferentiated cells (Fig. 1b). The marker genes of B cells were CD79A, CD19, MS4A1 (Fig. S1c); T cells were highly expressed in TRAC, CD2, CCL5, CD7, CD3D, CD3E (Fig. 1c); Colonocytes were highly expressed in AQP8, CEACAM7 and GUCA2A (Fig. S1d); enteroendocrine cells were highly expressed in TUBA1A, PTMS, PCSK1N, SCGN and CRYBA2 (Fig. S1e). Goblet cells highly expressed ANO7, MLPH, B3GNT6, SPINK4, ITLN1 and MUC2 (Fig. S1f); innate lymphoid cells highly expressed CD83, CD74, HLA-DPB1, HLA-DQB1 and HLA-DRA (Fig. S1g); mast cells highly expressed TPSB2, KIT, SLC18A2 and TPSAB1 (Fig. S1h); undifferentiated cells highly expressed CDCA7, CFTR, CLDN15, ADH1C and RARRES2 (Fig. 1d, Fig. S1i). We found that in ulcerative colitis tissues, they contained a large number of B cells, mast cells and T cells, which may be associated with lymphocytic infiltration of inflammatory tissues (Fig. 1e, f, Fig. S1j). In addition, the proportion of colonic cells in inflammatory tissues was much less than that of paraneoplastic tissues as well as normal tissues, which may be related to the destruction of inflammatory cell infiltration.

To elucidate the interactions between different cell types and to identify target cells that play an important role in inflammation progression, we used the CellChat package (Version 1.0.0) for cell communication analysis [14]. The results showed that mast cells, colon cells, and cupped cells interact more extensively with other cell types, in the tissue. The above analysis indicates that infiltration of inflammatory cells is an important pathological feature of ulcerative colitis and has an important association with colonocytes as well as cupped cells, implying the possibility that inflammatory cells have an important link in the progression of inflammation as well as prognosis.

### Identification of diagnostic model gene of ulcerative colitis

**Fig. 2:**
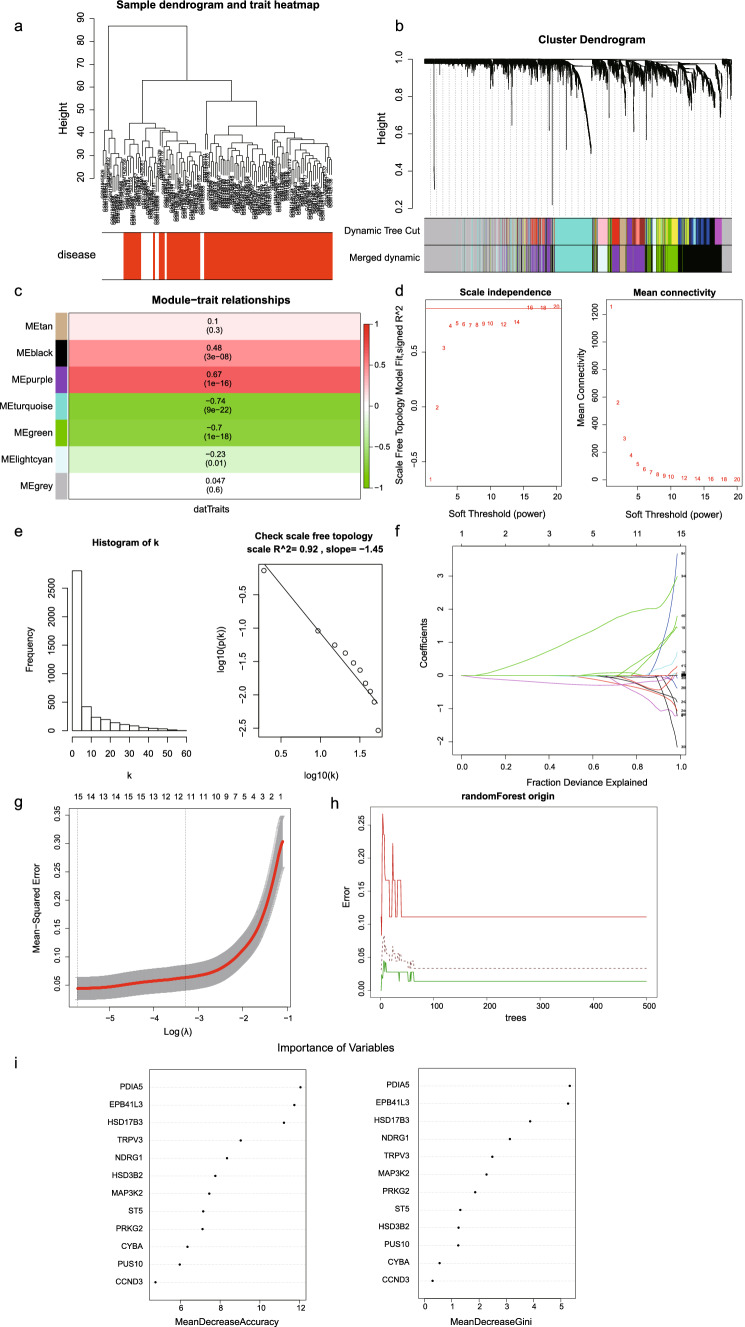
**a, b** Trait heatmap and sample dendrogram. The colors represent clinical traits. Red represents UC patients, white represents normal. **c** The correlation and *p* value of each module with the disease. The turquoise module has the highest correlation with the disease, and the *p* value is meaningful. Red indicates positive correlation with the disease, and green indicates negative correlation with the disease. **d** Left panel: scale-free topology fitting index of soft threshold power. Right panel: average connectivity of soft threshold power. **e** The selected soft threshold meets the non-scale requirement, and the non-scale *R*^2^ = 0.92. **f** Ten-time cross-validation for tuning parameter selection in the LASSO model. **g** LASSO coefficient profiles of the hub gene. A vertical line is drawn at the value chosen by 12-fold cross-validation. **h** The black line represents the mean error rate. **i** Rank the genes that contribute the most to the model, among which the first five genes are EPB41L3, HSD17B3, NDRG1, PDIA5, TRPV3

We performed weighted correlation network analysis (WGCNA) on deg of GSE75214 and single cells using the R package WGCNA [15]. WGCNA was used to construct gene co-expression networks to classify all genes into biogenic modules based on average linkage hierarchical clustering and further identify genes associated with UC diseases (Fig. 2a, b) To ensure that the network was scale-free, we chose a soft threshold of 16 and verified the validity of this soft threshold (Fig. 2d, e). The marker genes were divided into 7 modules, among which the turquoise module being the most associated with the occurrence of disease (Fig. 2c), so we selected the genes of turquoise module as hub genes. Further Lasso regression analysis was performed on these hub genes to obtain 12 disease-associated genes (Fig. 2f, g). We used the randomForest package to build a prediction model for the training set containing the 12 genes and found that the prediction accuracy of the training set (GSE75214) in random forest was 96.67%. Meanwhile, the prediction accuracy of the internal validation set was 86.21%. The model approached stability when the random forest tree reached 75 (Fig. 2h). We ranked the genes according to their contributions to the model and selected the top 5 genes that contributed the most to the model as model genes (Fig. 2i).

### Validation of model genes

**Fig. 3:**
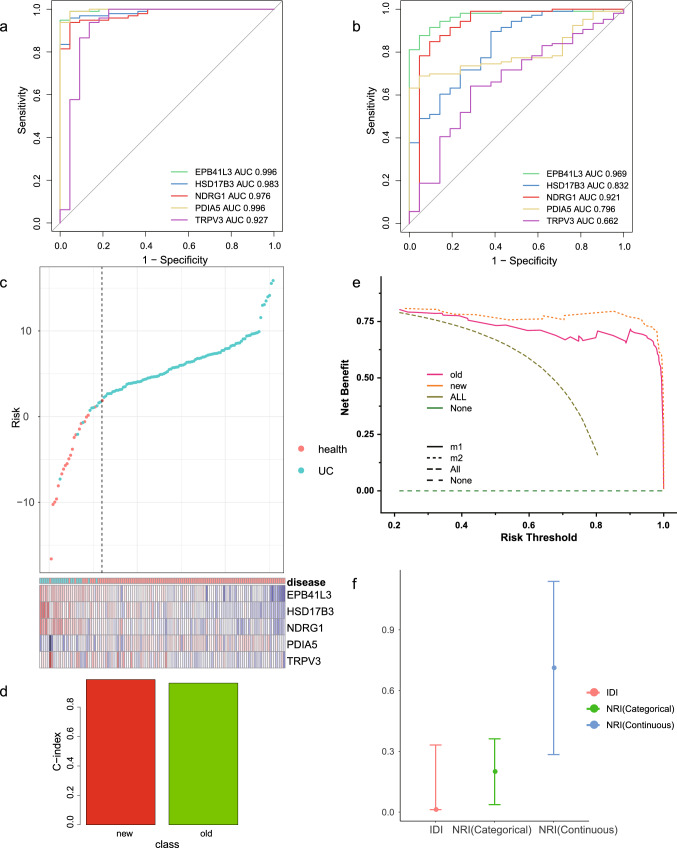
**a** AUC area of the five genes in the training set. **b** AUC area of the three genes in the external validation set. **c** Risk heat map, red indicates healthy patients, blue indicates UC patients, the left side of the dashed line is the low-risk group and the right side is the high-risk group. **d** Red is the C-index of the new model and green is the C-index of the old model. **e** Orange is the new model curve and red is the old model curve. **f** The upper and lower limits are 95 percent confidence intervals, and the middle point is the new model NRI/IDI improvement value

To confirm the independent diagnostic impact of individual genes, the accuracy of genes on diagnosis was demonstrated by ROC in both the internal training set and the external validation set. The AUCs in both the training set were greater than 0.90 (Fig. 3a) and the AUCs in the external validation set were greater than 0.66 (Fig. 3b), suggesting that these model genes have significant significance for the diagnosis of the disease and are highly likely to be closely associated with the lesions of ulcerative colitis, as well as being potential diagnostic factors. The risk plot based on the external validation set demonstrates the prevalence from low to high risk and the expression of model genes (Fig. 3c).

We compared the stability of the old and new (CCR7, CXCL10, CXCL9, IDO1, MMP9, VCAM1) (https://doi.org/10.3389/fphys.2019.00662) diagnostic models in an external validation set, and the C-index was used to measure the diagnostic accuracy of the old and new models. The results showed that the new model was more capable of diagnosing the disease than the old model (Fig. 3d). The Decision curve analysis indicates that the new model provides greater model’s net clinical benefit in disease diagnosis compared to the old model. (Fig. 3e) The new model significantly improved the diagnostic accuracy compared to the old model, NRI (Categorical: 0.1995, 95% CI 0.037–0.3619, *p* = 0.0161; Continuous: 0.7116, 95% CI 0.2842–1.139, *p* = 0.0011) and IDI (0.1717, 95% CI 0.012–0.3313, *p* = 0.035) (Fig. 3f).

### Functional characteristics of inflammatory tissue

**Fig. 4:**
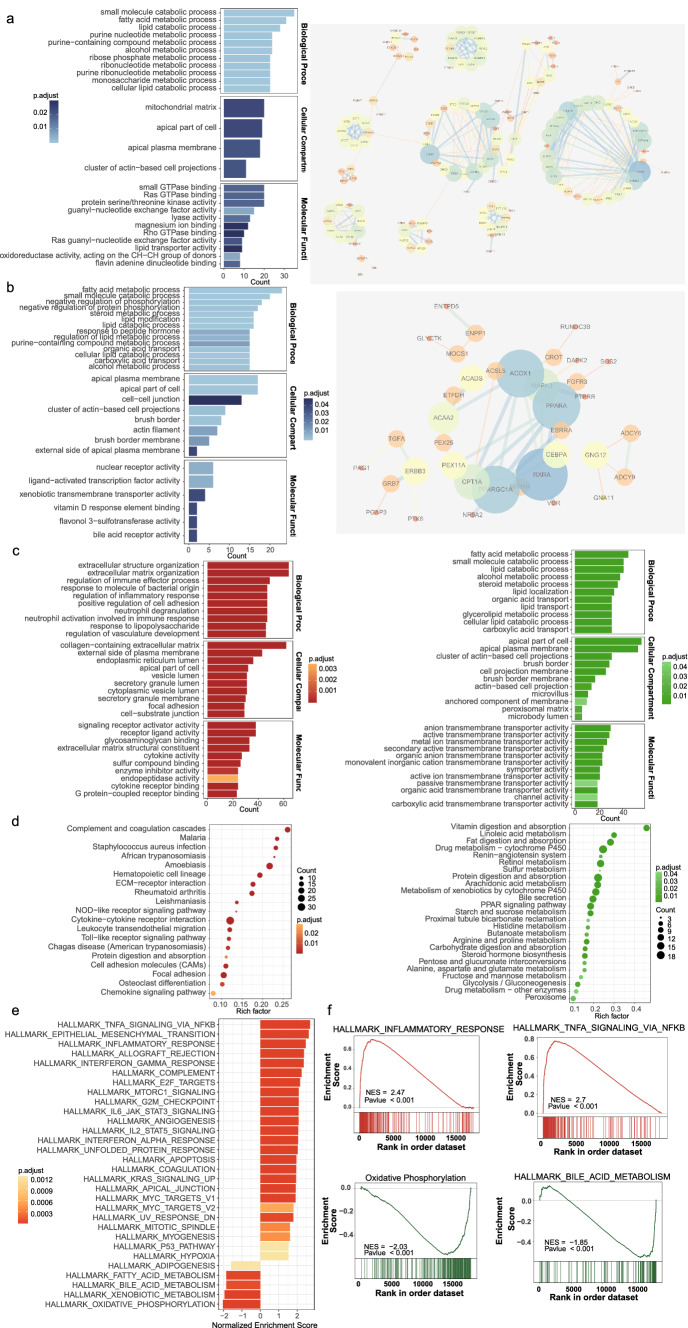
**a** The left panel showed the results of GO analysis of HSD17B3 positively related genes; the right panel showed the significant interactions of HSD17B3 positively related genes. **b** The left panel showed the results of GO analysis of NDRG1 positively related genes; the right panel showed the significant interactions of NDRG1 positively related genes. **c** The left panel showed the results of GO analysis for upregulated genes in UC samples; the right panel showed the results of GO analysis for downregulated genes in UC samples. Upregulated and downregulated genes were obtained from differential analysis. **d** The left panel showed the results of KEGG analysis of upregulated genes in UC samples, and the right panel showed the results of KEGG analysis of downregulated genes in UC samples. **e** Results of GSEA analysis of UC samples. **f** GSEA results of inflammatory response, Tnfα signaling via NFκB, oxidative phosphorylation and xenobiotic metabolism

We, respectively, performed correlation analysis for the five genes in the datasets GSE72514 and GSE87473, and took the intersection of genes with correlation coefficients greater than 0.5 as positively correlated genes. Then, we conducted GO analysis for the positively correlated genes. Our study showed that EPB41L3 was closely associated with lipid-related metabolic pathways such as fatty acid metabolic process, lipid catabolic process, regulation of lipid metabolic process, steroid metabolic process, lipid localization, lipid transport, and lipid transporter protein activity (Fig. S2a). PDIA5 was associated with neutrophil degranulation, activation of neutrophils involved in immune response, mitosis, regulation of cell cycle phase transition, and ATPase activity (Fig. S2b). HSD17B3 was closely associated with fatty acid metabolic processes, lipid catabolic processes, ribonucleotide metabolic processes and monosaccharide metabolic processes, oxidoreductase activity and lipid transporter protein activity (Fig. 4a). NDRG1 was associated with fatty acid metabolic processes, steroid metabolic processes, carboxylic acid transport and organic acid transport (Fig. 4b). TRPV3 was associated with glycerolipid metabolic processes (Fig. S2c). In summary, our model genes had important connections in lipid metabolism, especially fatty acid metabolism and lipid metabolism, and immune response.

GO analysis of upregulated genes in inflammatory tissues suggested that immune response, inflammatory response, neutrophil degranulation and activation, and vascular development were significantly upregulated, and molecules such as cytokines and G protein-coupled receptors were also activated (Fig. 4c). KEGG results suggested that Toll-like receptor signaling pathways, leukocyte transendothelial migration, cytokine-cytokine receptor interaction, NOD-like receptor signaling pathway, and chemokine signaling pathway were also obviously upregulated (Fig. 4d). GSEA analysis suggested that epithelial–mesenchymal transition, TNFA signaling by NFKB, inflammatory response, MTORC1 signaling pathway, G2M checkpoint, IL6 JAK STAT3 signaling pathway, IL2 STAT5 signaling pathway, KRAS signaling pathway, and hypoxia were also in upregulated state (Fig. 4e, f). In contrast, oxidative phosphorylation, allogeneic metabolism, fatty acid metabolism, and adipogenesis were in a downregulated state.

In conclusion, there was a significant similarity for the function of model genes and the metabolic profile of ulcerative colitis, which also explained the rationality and biological mechanism of model genes for the diagnosis of ulcerative colitis.

### Functional characteristics of drug-sensitive tissue

**Fig. 5:**
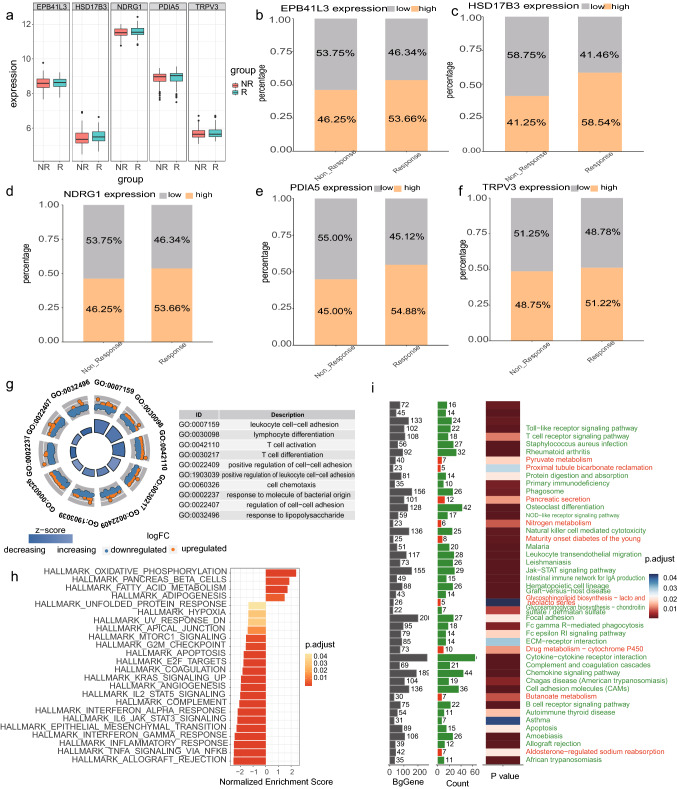
**a** Model genes expression in NR group samples and R group samples. Histograms depicted the expression of **b** EPB41L3, **c** HSD17B3, **d** NDRG1, **e** PDIA5, and **f** TRPV3 in NR group samples and R group samples. **g** Results of GO analysis in NR group samples and R group samples. **h** Results of GSEA analysis in NR group samples and R group samples. **i** Results of KEGG analysis in NR group samples and R group samples. The first column indicated the number of background genes, the second column indicated the number of enriched genes, and the third column indicated the *p* value of the pathways

Expression analysis of model genes revealed that it had higher expression in R group samples compared with NR group samples (Fig. 5a). In order to further explore the relationship and mechanism of model genes with drug sensitivity, we performed functional analysis based on the results of differential analysis between NR group samples and R group samples. The results of GO analysis indicated that lymphocyte differentiation, T cell activation, T cell differentiation, cell adhesion, and cell chemotaxis pathways were downregulated in drug-sensitive samples (Fig. 5g). KEGG results showed that drug metabolism—cytochrome P450 and nitrogen metabolism were upregulated in drug-sensitive samples, while T cell receptor signaling pathway, Toll-like receptor signaling pathway, NOD-like receptor signaling pathway, Jak-STAT signaling pathway, leukocyte transendothelial migration, ECM-receptor interaction, FcεRI signaling pathway, Fc γ R-mediated phagocytosis, chemokine signaling pathway, cytokine–cytokine receptor interaction, and B cell receptor signaling pathway were downregulated (Fig. 5i). The GSEA results suggest that adipogenesis, fatty acid metabolism, and oxidative phosphorylation are activated in drug-sensitive samples. In contrast, the inflammatory response, epithelial–mesenchymal transition, IL6 JAK STAT3 signaling, G2M checkpoint and MTORC1 signaling pathways were inhibited. GSEA results suggested that adipogenesis, fatty acid metabolism, and oxidative phosphorylation were in an activated state in drug-sensitive samples. On the contrary, inflammatory response, epithelial–mesenchymal transition, IL6 JAK STAT3 signaling, G2M checkpoint and MTORC1 signaling pathways were inhibited (Fig. 5h).

Our study demonstrated that samples with high-risk score were more sensitive to drugs and had greater efficacy than low-risk samples (Fig. 5b–f). Overall, our model genes might activate lipid metabolism and inhibit T cells, inflammatory response and tumor-related pathways to stimulate the body’s sensitivity to drugs (it possibly related to promoting drug uptake and inhibiting drug consumption, which increased drug residence time in the body). Thus, our model was not only beneficial in assisting diagnosis, but also had implications for predicting drug efficacy.

### High abundance of TIICs found with ssGSEA or CIBERSORT

**Fig. 6:**
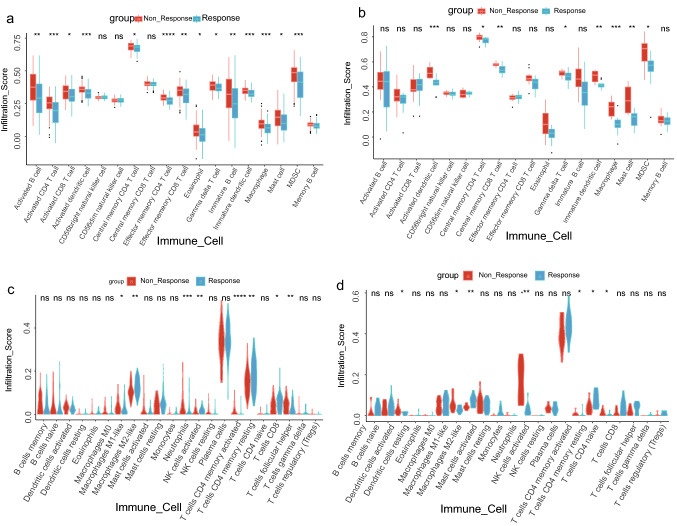
**a, b** Box plot based on ssGSEA shows the composition of 18 immune cells between R tissues and NR tissues in the GSE92415 and GSE12251 cohorts. The blue box plot indicates R individual, and the red violin box indicates non-response patients, the upper and lower ends of the box indicate the interquartile range of values, the line in the box indicates the median value, and the asterisk indicates the statistical *p* value (*p < 0.05; **p < 0.01; ***p < 0.001). **c, d** Violin plot based on CIBERSORT shows the composition of 22 immune cells between R tissues and NR tissues in the 2 cohorts. The blue box plot indicates R individual, and the red violin box indicates non-response patients, the upper and lower ends of the box indicate the interquartile range of values, and the asterisk indicates the statistical *p* value (*p < 0.05; **p < 0.01; ***p < 0.001)

Based on the GSE92415 and GSE12251 datasets, the ssGSEA algorithm was used to evaluate 18 immune signatures between NR group samples and R group samples. The results showed that the NR group samples had higher levels of activated dendritic cells, central memory CD4 T cells, Gamma delta T cells, immature dendritic cells, macrophage, mast cells, and myeloid-derived suppressor cell (MDSC) (*p* value < 0.05). There was no significant difference between CD56bright natural killer cell, CD56dim natural killer cell, Central memory CD8 T cell (Fig. 6a, b). Therefore, the intensity of immune cell infiltration was higher in the drug-resistant group than in the drug-sensitive group.

The proportion of 22 immune cell types was calculated by CIBERSORT. We performed the immune assessment with the GSE92415 and GSE12251. We compared the proportions of immune cells in the NR group samples and R group samples. NR group samples had higher proportions of Macrophages M1, Neutrophils, T cells CD4 memory activate. Neutrophils perpetuate intestinal inflammation and recruit other immune cells, releasing multiple inflammatory mediators making the process worse. The macrophages present in the inflamed tissue in a high concentration are known as M1-like classically activated macrophages. Gene expression profile of M1-like macrophages exhibit high levels of pro-inflammatory cytokines like tumour necrosis factor-a (TNF-a), monocyte chemoattractant protein-1 (MCP-1), Interleukin-6 (IL-6) and inducible nitric oxide synthase (iNOS) [16]. Present in intestinal tissue, Microbiota-reactive CD4+ T cells were mainly of a memory phenotype. And These cells could stimulated intestinal stromal and epithelial cells via tumor necrosis factor [17]. Therefore, T cells CD4 memory are activated in NR group to promotes inflammatory responses including the expression of inflammatory cytokines. Macrophages M2, T cells CD4 memory resting were higher in R group samples (Fig. 6c, d). Macrophages present in relatively high concentration in normal are known as alternatively activated M2-like macrophages characteristically expressing high levels of IL-10, YM1, macrophage and granulocyte inducer-form 1(MgI1), arginase-I to name the important ones and are actively involved in tissue remodelling and repair [16]. To summarize, the intensity of immune cell infiltration was higher in NR group samples. Thus, the immune microenvironment was important for drug sensitivity. This suggested that model genes might affect the drug sensitivity by impacting immune cells.

## Discussion

The clinical diagnosis of ulcerative colitis hitherto relies on clinical symptoms, endoscopic examination, and relevant etiological examinations [18]. According to previous studies, perinuclear anti-neutrophil cytoplasmic antibody (PANCA) combined with anti-yeast antibody (ASCA) could be helpful to diagnose ulcerative colitis. However, because of its low sensitivity, it is still not promoted for clinical application [19]. In addition, C-reactive protein (CRP), fecal calprotectin, and fecal ferritin also have certain significance for the diagnosis of ulcerative colitis, but they are susceptible [20]. Hence, it is necessary to find the diagnostic markers with high specificity, high sensitivity, convenience and economy. In this study, we constructed a clinical diagnostic model consisting of 5 biomarkers. Besides being used for clinical diagnosis, we also extended it to the prediction of anti-TNF-ɑ drug efficacy.

Single-cell sequencing is a technique, capturing mRNA transcripts in the specific cells and converting them into libraries for sequencing, which allows researchers more precisely to identify suspicious cells [21]. We used ulcerative colitis single-cell data to identify cells with an important role in pathogenesis. We found that infiltration of inflammatory cells is an essential pathological feature of ulcerative colitis. Mast cells, colonocytes and cupped cells interacted more extensively with other cell types, which suggested that mast cells played an important role in inflammatory tissues and the tissue cells of UC samples might have an abnormal functional state.

Previous studies have suggested the involvement of lipid metabolism in the pathogenesis of inflammatory bowel disease [22]. In a metabolomics study of inflammatory bowel disease, which includes serum samples from 40 patients with inflammatory bowel disease as well as 20 patients without inflammatory bowel disease, it is suggested that the levels of lipid metabolism-related metabolites, tricarboxylic acid cycle intermediates, and amino acids are significantly decreased in patients with inflammatory bowel disease [23]. The same biologic feature was found in our study: In UC samples, there was an obvious metabolic disturbance. For instance, oxidative phosphorylation, fatty acid metabolism and adipogenesis were inhibited. This is similar to the findings of previous studies [24]. Notably, we also found that tumor-related pathways such as epithelial–mesenchymal transition, TNFA signaling by NFKB, inflammatory response, G2M checkpoint, IL6 JAK STAT3 signaling pathway, IL2 STAT5 signaling pathway, KRAS signaling pathway and hypoxia were upregulated in ulcerative colitis tissues. In a way, this suggested that ulcerative colitis had malignant potential, which highlighted the importance of early diagnosis and early treatment for the clinical management of ulcerative colitis.

The analysis of positively related genes of model genes revealed that five models were closely related to lipid metabolism, mitosis and regulation of cell cycle. EPB41L3 was closely related to fatty acid metabolism, lipid metabolism, and steroid metabolism metabolic pathways. In addition, it has been shown that EPB41L3 can also inhibit cell growth by inducing apoptosis and cell cycle arrest. Positive correlation between PDIA5 and immune infiltrating cells, immune related pathways, inflammatory activities, and other immune checkpoint members in gliomas [25]. And our study demonstrates that PDIA5 was associated with neutrophil degranulation, activation of neutrophils involved in immune response, mitosis, regulation of cell cycle phase transition, and ATPase activity in UC. Therefore, we speculate that PDIA5 may promote immune cell-related inflammatory responses by activating neutrophils. PDIA5 was associated with neutrophil activation, mitotic processes. HSD17B3 was associated with fatty acid metabolism, lipid metabolism, and ribonucleotide metabolism. NDRG1 is a cytoplasmic protein involved in hormone response, stress response, differentiation and cell growth in p53-mediated apoptosis and activation of caspases. Some studies propose that NDRG1 may play a key role in the development of inflammatory bowel disease and may serve as specific therapeutic targets for the treatment of inflammatory bowel disease. In our study, NDRG1 was found to be associated with fatty acid metabolic processes, steroid metabolic processes, carboxylic acid transport and organic acid transport. TRPV3 was linked to glycerolipid metabolic processes. TRPV3 has been shown to be associated with disease activity in the study by Joel J. Toledo Mauriño et al. TRPV3 has been proposed as a relevant mechanism of gastrointestinal inflammation. Since T cell activation by mitogen or TCR stimulationis dependent on TRPVs and T cell activation promotes inflammatory response, Changes in TRPV3 expression is a mechanism to reinforce pro-inflammatory immune responses downstream of T cell activation [26]. Taken together, our model genes have important implications in energy metabolism, immune response and cell growth and proliferation. This also explained the rationale and biological mechanism of model genes for the diagnosis of ulcerative colitis at the genetic level.

The level of body energy expenditure is significantly correlated with the disease activity in patients with inflammatory colitis. Patients with moderate to severe ulcerative colitis are hypermetabolized, and induction therapy can significantly alter the body’s energy metabolism. We explored the immune and metabolic characteristics of the R group samples and the NR group samples. Our results suggested that the samples in the R group were at high metabolic levels, such as adipogenesis, fatty acid metabolism, and oxidative phosphorylation. While, the immune-related pathways, tumor-related pathways (such as epithelial–mesenchymal transition, IL6 JAK STAT3 signaling, and G2M checkpoint) and inflammation-related pathways were downregulated. Immunological analysis of the R group samples and NR group samples revealed that the degree of immune cell infiltration was generally higher in the NR group samples. In conclusion, ulcerative colitis with high metabolism and low malignancy showed a higher degree of benefit from monoclonal antibody drug therapy against TNF-α.

In our study, we had developed a diagnostic model with greater specificity and sensitivity, which performed better than the biomarkers proposed in previous studies. Our model was also suggestive for clinical drug usage and prediction of drug efficacy. However, its clinical practicability needed to be further substantiated by more clinical researches. Our study fully explained the principle and mechanism of action of the diagnostic model through the metabolism, immunity and cell growth and proliferation of the model genes from multiple perspectives, which provided significant theoretical support for clinical application.

## Conclusion

In this study, five biomarkers were screened using single-cell data and transcriptomic data for the construction of clinical diagnostic and drug sensitivity prediction models. The model has better specificity, sensitivity and stability, and is useful for drug sensitivity prediction, which will have higher value for clinical research and application (Fig. S3 Created with BioRender.com). EPB41L3 encodes protein 4.1B, which is a membrane skeletal protein that belongs to the protein 4.1 family. Protein 4.1B/DAL-1 is localized to sites of cell-cell contact and functions as an adapter protein, linking the plasma membrane to the cytoskeleton or associated cytoplasmic signaling effectors and facilitating their activities in various pathways. Protein 4.1B/DAL-1 is involved in various cytoskeleton-associated processes, such as cell motility and adhesion [27]. The results of GO analysis indicated that lymphocyte differentiation, T cell activation, T cell differentiation, cell adhesion, and cell chemotaxis pathways were down-regulated in drug-sensitive samples (Fig. 5g). So EPB41L3 might reduce protein 4.1B expression to downregulate T cell activation, T cell differentiation, cell adhesion, and cell chemotaxis pathways to suppress T cell function in inflammation. PDIA5 was associated with neutrophil degranulation, activation of neutrophils involved in immune response, mitosis, regulation of cell cycle phase transition, and ATPase activity (Fig. S2b). Therefore, possibly PDIA5 downregulates neutrophil degranulation,and activation of neutrophils involved in immune response to reduce the inflammatory response. Moreover, our study demonstrates that EPB41L3, TRPV3, NDRG1, HSD17B3 might upregulate lipid metabolism, especially fatty acid metabolism and lipid metabolism, which is favorable for the reduction of inflammation. Macrophage polarization is believed to play an important role in inflammation and host defense mechanisms through a dynamic process [28]. Lipid metabolism has a key role in regulating macrophage functions. Signals that drive macrophage activation (e.g., to an inflammatory state that regulates host defense) impinge on metabolic-sensing pathways to coordinate shifts in lipid metabolism. Lipids are a source of energy for macrophages, and provide precursors for bioactive lipids and components of cellular membranes. Lipids also regulate signal transduction and gene regulation during macrophage activation [29]. Assessed by functional enrichment analysis, we found that HSD17B3, NDRG1, PDIA5, and TRPV3 have important links with lipid metabolism and fatty acid metabolism. From this, we infer that among macrophage polarization, lipid metabolism and our model genes, there is a complex regulatory network that needs to be further explored.

## Supplementary Information

Below is the link to the electronic supplementary material.

Supplementary file1 **Supplementary Fig. 1 a** Tsne diagram of all cells, where cells were divided in 16 clusters. **b** Batch effects in Single-cell data. The left panel showed Tsne diagram of all cells before removing batch effects; The right panel showed Tsne diagram of all cells before removing batch effects. The marker genes expression of **c** B cells, **d** colonocytes, **e** enteroendocrine cells, **f** goblet cells, **g** innate lymphoid cells, **h** mast cells and **i** undifferentiated cells. **j** The distribution of all cell types in samples (JPG 7226 KB)

Supplementary file2 **Supplementary Fig. 2 a** The results of GO analysis of EPB41L3 positively related genes. **b** The results of GO analysis of PDIA5 positively related genes. **c** The results of GO analysis of TRPV3 positively related genes (JPG 5485 KB)

Supplementary file3 (PNG 2912 KB)

## References

[1] Ananthakrishnan AN (2015). Epidemiology and risk factors for IBD. Nat Rev Gastroenterol Hepatol.

[2] Loftus EV (2004). Clinical epidemiology of inflammatory bowel disease: incidence, prevalence, and environmental influences. Gastroenterology.

[3] Kornbluth AA (1993). Meta-analysis of the effectiveness of current drug therapy of ulcerative colitis. J Clin Gastroenterol.

[4] Kornbluth A, DB Sachar, and Practice Parameters Committee of the American College of Gastroenterology (2010). Ulcerative colitis practice guidelines in adults: American college of gastroenterology, practice parameters committee. Am J Gastroenterol.

[5] Hwang B, Lee JH, Bang D (2018). Single-cell RNA sequencing technologies and bioinformatics pipelines. Exp Mol Med.

[6] Mitsialis V (2020). Single-cell analyses of colon and blood reveal distinct immune cell signatures of ulcerative colitis and Crohn’s disease. Gastroenterology.

[7] Gautier L (2004). affy–analysis of Affymetrix GeneChip data at the probe level. Bioinformatics.

[8] Irizarry RA (2003). Exploration, normalization, and summaries of high density oligonucleotide array probe level data. Biostatistics.

[9] Tibshirani R (1997). The lasso method for variable selection in the Cox model. Stat Med.

[10] Vickers AJ, Elkin EB (2006). Decision curve analysis: a novel method for evaluating prediction models. Med Decis Making.

[11] Newman AM (2015). Robust enumeration of cell subsets from tissue expression profiles. Nat Methods.

[12] Bindea G (2013). Spatiotemporal dynamics of intratumoral immune cells reveal the immune landscape in human cancer. Immunity.

[13] Aran D (2019). Reference-based analysis of lung single-cell sequencing reveals a transitional profibrotic macrophage. Nat Immunol.

[14] Jin S (2021). Inference and analysis of cell-cell communication using CellChat. Nat Commun.

[15] Langfelder P, Horvath S (2008). WGCNA: an R package for weighted correlation network analysis. BMC Bioinform.

[16] Moreira Lopes TC, Mosser DM, Gonçalves R (2020). Macrophage polarization in intestinal inflammation and gut homeostasis. Inflamm Res.

[17] Hegazy AN, West NR, Stubbington MJT, Wendt E, Suijker KIM, Datsi A, This S, Danne C, Campion S, Duncan SH, Owens BMJ, Uhlig HH, McMichael A, Bergthaler A, Teichmann SA, Keshav S, Powrie F (2017). Circulating and tissue-resident CD4+ T cells with reactivity to intestinal microbiota are abundant in healthy individuals and function is altered during inflammation. Gastroenterology..

[18] Kaenkumchorn T, Wahbeh G (2020). Ulcerative colitis: making the diagnosis. Gastroenterol Clin North Am.

[19] Linskens RK (2002). Evaluation of serological markers to differentiate between ulcerative colitis and Crohn’s disease: pANCA, ASCA and agglutinating antibodies to anaerobic coccoid rods. Eur J Gastroenterol Hepatol.

[20] Mosli MH (2015). C-reactive protein, fecal calprotectin, and stool lactoferrin for detection of endoscopic activity in symptomatic inflammatory bowel disease patients: a systematic review and meta-analysis. Am J Gastroenterol.

[21] Kolodziejczyk AA (2015). The technology and biology of single-cell RNA sequencing. Mol Cell.

[22] Koutroumpakis E (2016). Association between long-term lipid profiles and disease severity in a large cohort of patients with inflammatory bowel disease. Dig Dis Sci.

[23] Scoville EA (2018). Alterations in lipid, amino acid, and energy metabolism distinguish Crohn’s disease from ulcerative colitis and control subjects by serum metabolomic profiling. Metabolomics.

[24] Tan M (2020). Fatty acid metabolism in immune cells: a bioinformatics analysis of genes involved in ulcerative colitis. DNA Cell Biol.

[25] Zhang H, He J, Dai Z, Wang Z, Liang X, He F, Xia Z, Feng S, Cao H, Zhang L, Cheng Q (2021). PDIA5 is correlated with immune infiltration and predicts poor prognosis in gliomas. Front Immunol..

[26] Majhi RK, Sahoo SS, Yadav M, Pratheek BM, Chattopadhyay S, Goswami C (2015). Functional expression of TRPV channels in T cells and their implications in immune regulation. The FEBS J..

[27] Wang Z, Zhang J, Ye M, Zhu M, Zhang B, Roy M, Liu J, An X (2014). Tumor suppressor role of protein 4.1B/DAL-1. Cell Mol Life Sci..

[28] El-Arabey AA, Denizli M, Kanlikilicer P, Bayraktar R, Ivan C, Rashed M, Kabil N, Ozpolat B, Calin GA, Salama SA, Abd-Allah AR, Sood AK, Lopez-Berestein G (2020). GATA3 as a master regulator for interactions of tumor-associated macrophages with high-grade serous ovarian carcinoma. Cell Signal..

[29] Yan J, Horng T (2020). Lipid metabolism in regulation of macrophage functions. Trends Cell Biol..

