# Supplementary figures and images for: Single-cell and microarray chip analysis revealed the underlying pathogenesis of ulcerative colitis and validated model genes in diagnosis and drug response
Source: Hum Cell. 2022 Nov 29;36(1):132–45. doi: 10.1007/s13577-022-00801-6 (PMC9813122; doi:10.1007/s13577-022-00801-6)

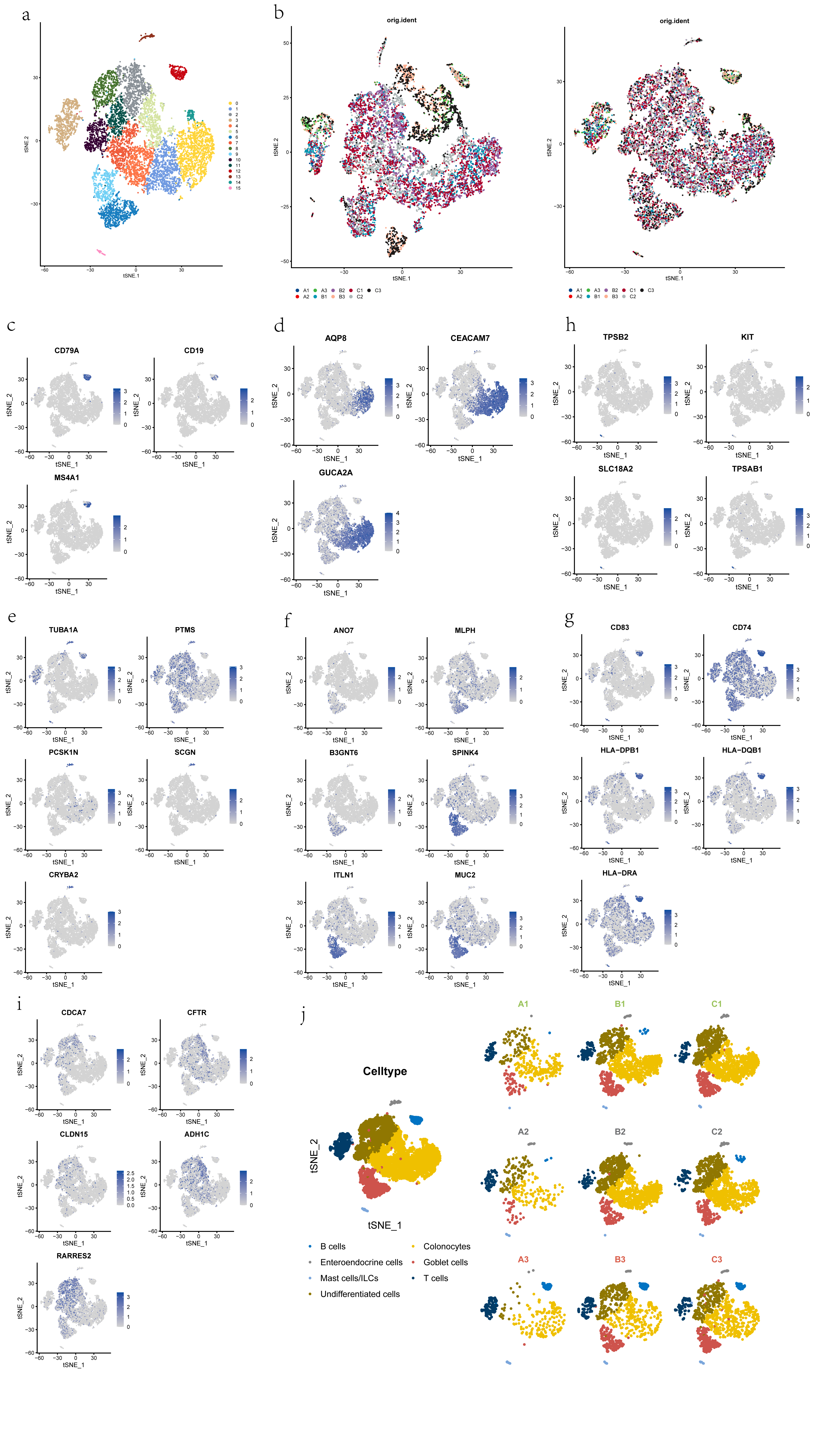

Supplement: Supplementary file 1 — Supplementary file1 Supplementary Fig. 1 a Tsne diagram of all cells, where cells were divided in 16 clusters. b Batch effects in Single-cell data. The left panel showed Tsne diagram of all cells before removing batch effects; The right panel showed Tsne diagram of all cells before removing batch effects. The marker genes expression of c B cells, d colonocytes, e enteroendocrine cells, f goblet cells, g innate lymphoid cells, h mast cells and i undifferentiated cells. j The distribution of all cell types in samples (JPG 7226 KB) [file 13577_2022_801_MOESM1_ESM.jpg]

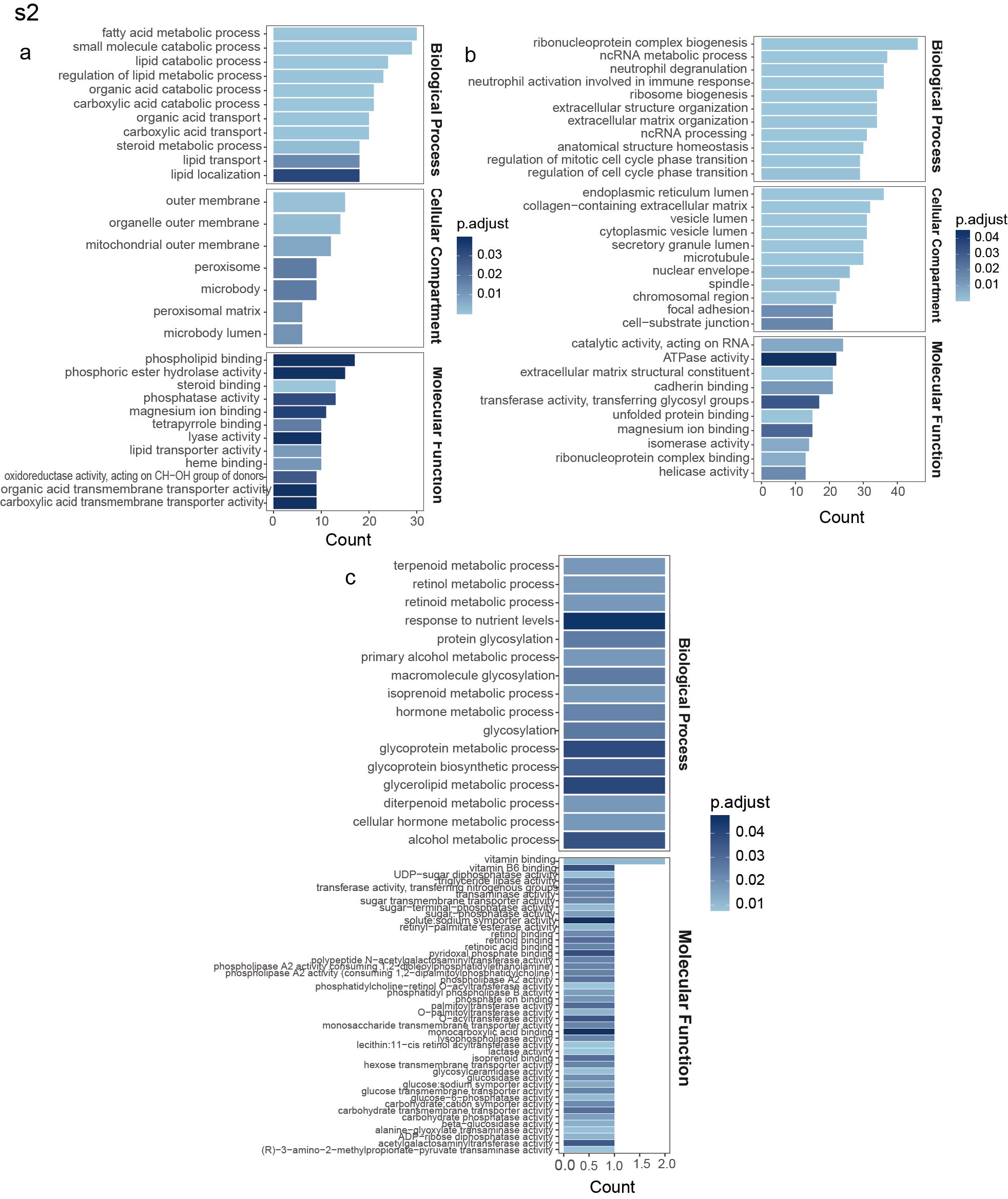

Supplement: Supplementary file 2 — Supplementary file2 Supplementary Fig. 2 a The results of GO analysis of EPB41L3 positively related genes. b The results of GO analysis of PDIA5 positively related genes. c The results of GO analysis of TRPV3 positively related genes (JPG 5485 KB) [file 13577_2022_801_MOESM2_ESM.jpg]

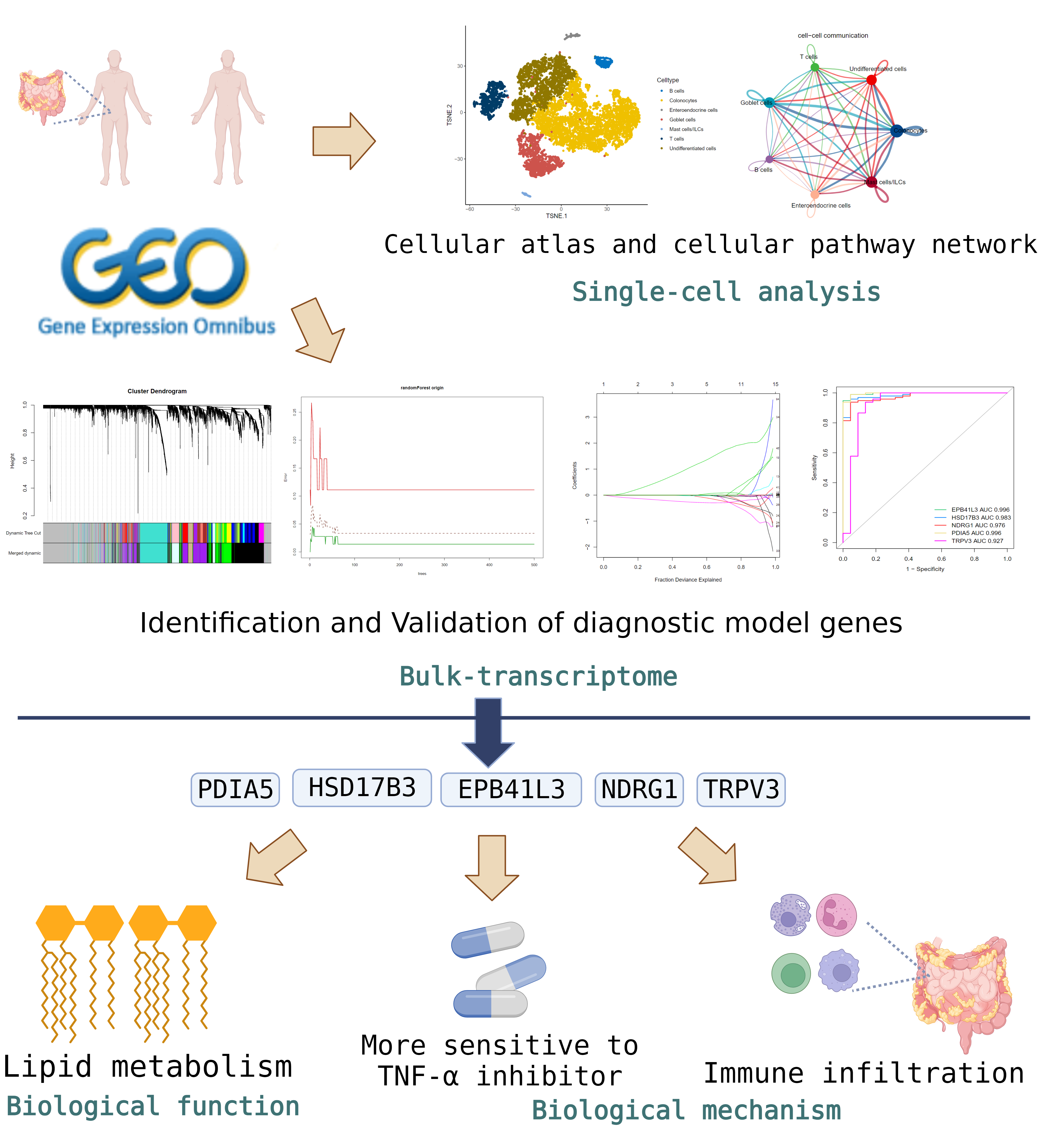

Supplement: Supplementary file 3 — Supplementary file3 (PNG 2912 KB) [file 13577_2022_801_MOESM3_ESM.png]
